# Unipolar memristive Switching in Bulk Negative Temperature Coefficient Thermosensitive Ceramics

**DOI:** 10.1371/journal.pone.0079832

**Published:** 2013-11-08

**Authors:** Hongya Wu, Kunpeng Cai, Ji Zhou, Bo Li, Longtu Li

**Affiliations:** 1 State key laboratory of new ceramic and fine processing, School of materials science and engineering, Tsinghua University, Beijing, China; 2 Advanced Materials Institute, Shenzhen Graduate School, Tsinghua University, Shenzhen, China; University of Nebraska-Lincoln, United States of America

## Abstract

A memristive phenomenon was observed in macroscopic bulk negative temperature coefficient nickel monoxide (NiO) ceramic material. Current-voltage characteristics of memristors, pinched hysteretic loops were systematically observed in the Ag/NiO/Ag cell. A thermistor-based model for materials with negative temperature coefficient was proposed to explain the mechanism of the experimental phenomena. Most importantly, the results demonstrate the potential for a realization of memristive systems based on macroscopic bulk materials.

## Introduction

Memristor (contraction of memory and resistor) was first proposed by Leon Chua in 1971, which is postulated as the forth fundamental passive circuit element alongside resistors, capacitors, and inductors [1,2]. Recently considerable interest has been attracted to the memristor including using memristors as nonvolatile memory devices[3-5], and applying memristors in neuromorphic circuits[6-8] and circuit design[9,10], etc. 

Since the memristor based on a thin film of TiO_2_ was first announced in 2008[11], many materials for memristor have been explored such as SrTiO_3-x_N_y_[12,13], Gd_2_O_3_[14], ZnO[15], ZrO_2_[16], VO_2_[17,18], etc. Devices are mostly fabricated in forms of nanoscale structures using nanoimprint lithography [19]. Panda et al have investigated the potential memristor behaviour of NiO films and the switching phenomena were explained using the rupture and formation of conducting filaments which is mostly used in memristive films[20]. However, little attention has been paid to the memristive behaviors in macroscale bulk materials, which is compatible with the state-of-art passive electronics technology. D. J. Kim has reported that a Kondo insulator Ce_3_Bi_4_Pt_3_ satisfies the necessary condition for a memristor which can be explained by the virtual thermal impedance arising from self heating[21].

In this work, memristive phenomenon was observed in a bulk Ag/NiO/Ag sandwich structure, a thermistor-based model with the consideration of the negative temperature coefficient (NTC) effect was proposed to explain the memristive mechanism.

## Methods

NiO powder was pressed into pellets in diameter of 10 mm under a pressure of 4 MPa. The pellets were sintered in a Nabertherm furnace (LTH 08/17, Nabertherm, Germany) at 1300 °C for 2h. A NiO sample with a diameter of 8.36 mm, thickness of 0.5 mm, and weight of 0.16 g was obtained. For electrical property measurements, electrodes were fabricated on opposite pellet faces from Ag paste. The current-voltage (I-V) characteristics were measured and memristive switching was observed in the devices. The dependences of the *I-V* plots on voltage, voltage scan rate, temperature, size of the samples were investigated. To explore the effect of size of the samples on *I-V* plots, a series of samples with different thicknesses were constructed. 

The X-ray diffraction (XRD) was recorded using a diffractometer (D/Max B, Rigaku, Japan). An impedance analyzer (HP4192A, Agilent Technologies, USA) was used to measure the resistance of the sintered samples, while a digitally controlled temperature chamber (2300, Delta Design, USA) was used to control the temperature. The *I-V* characteristics were measured by a power device analyzer/curve tracer (B1505, Agilent Technologies, USA). 

## Results and Discussion

**Figure 1:**
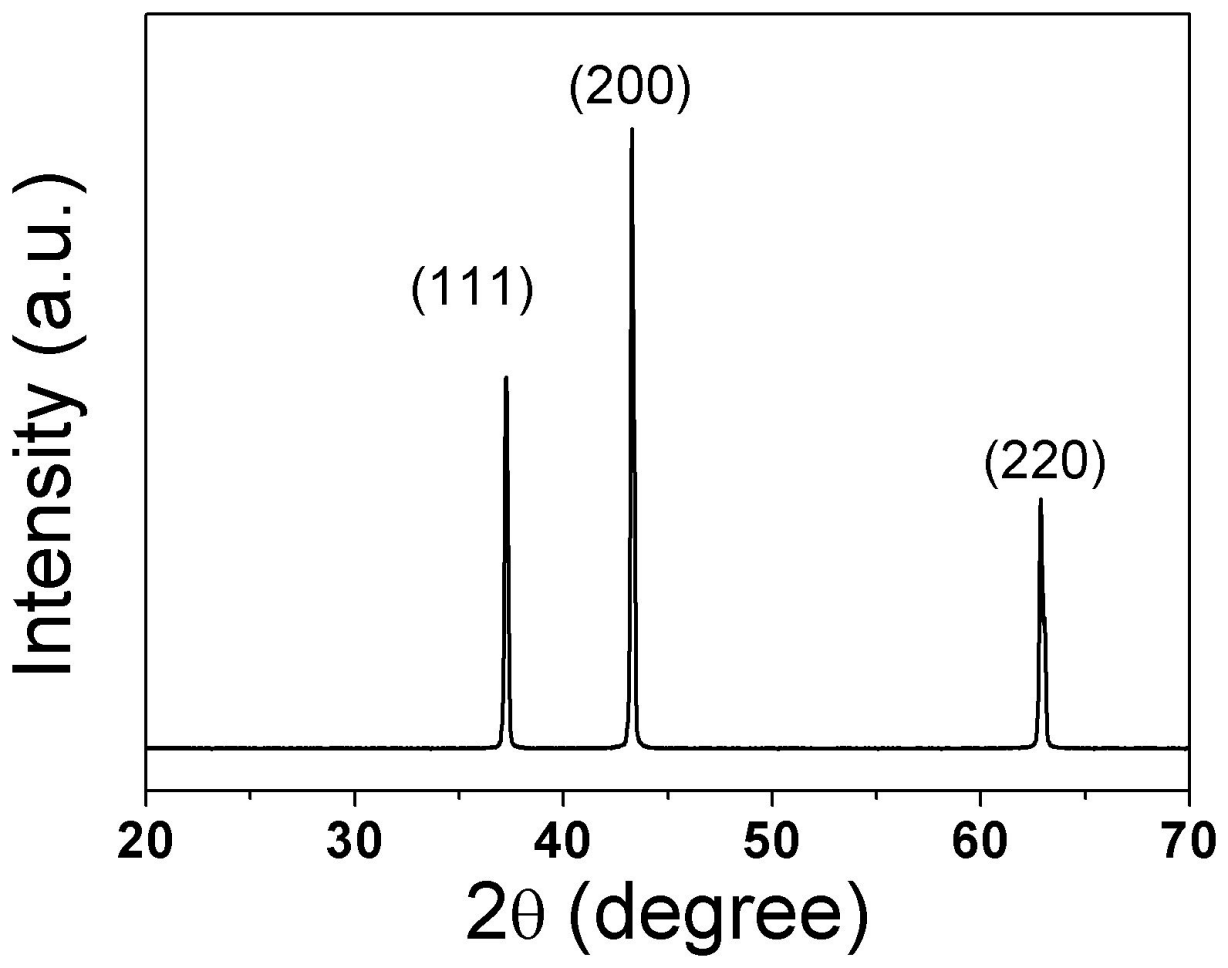
XRD patterns of NiO sintered at 1300 °C.

The XRD analysis was used for the phase identification as illustrated in Figure 1. The patterns shown in the spectra are all indexed to NiO cubic phase with Fm-3m as space group, and no obvious secondary phase can be detected in the samples, which indicates that NiO powders have been sintered to NiO polycrystalline ceramics with no chemical reaction.

**Figure 2:**
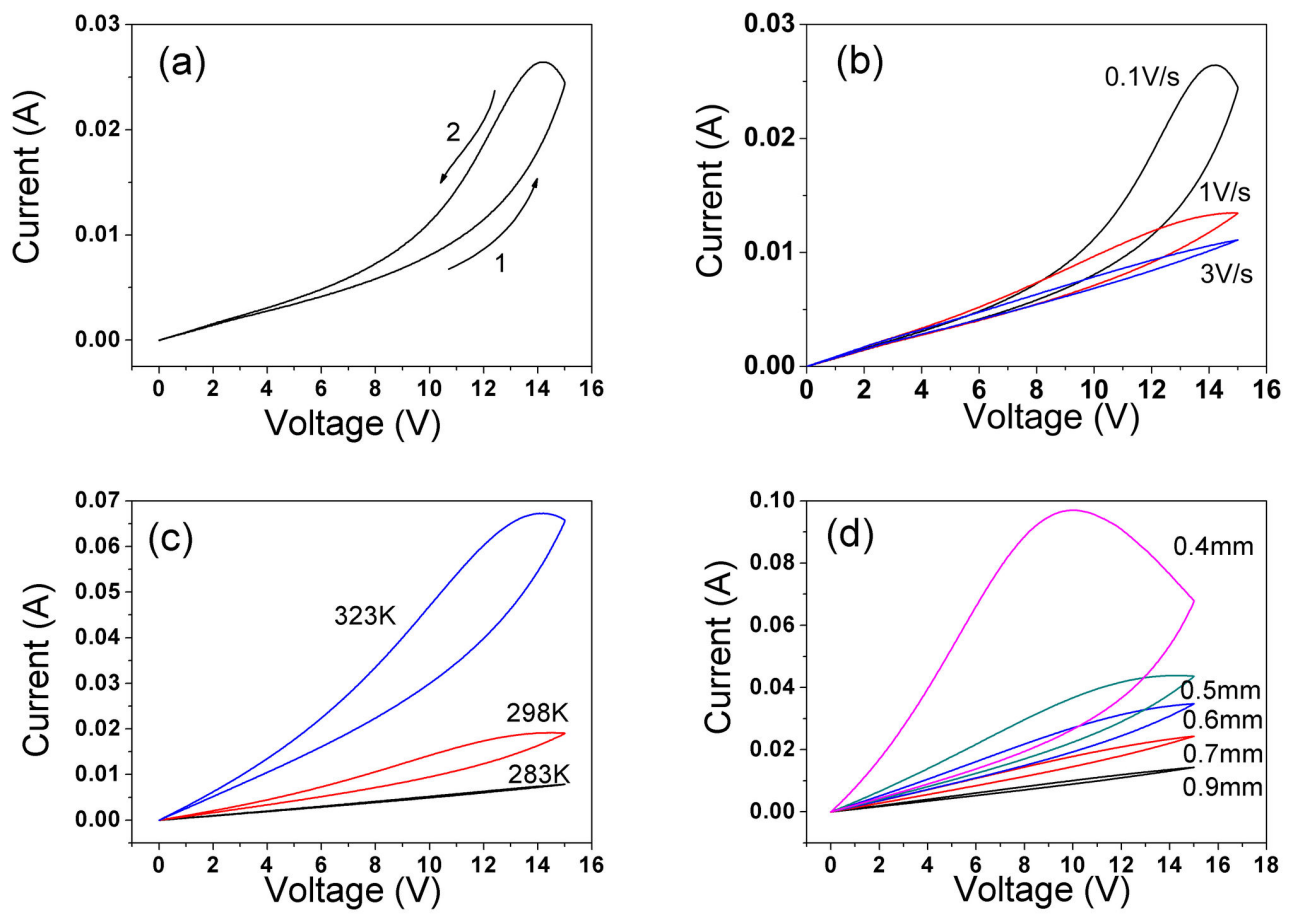
*I-V* characteristics of NiO for V_max_ =15 V (a) measured at voltage scan rate of 0.1V/s; (b)measured at voltage scan rate of 0.1, 1, and 3 V/s; (c) at different temperatures: 283, 298 and 323 K; (d) with different sample thicknesses: 0.4, 0.5, 0.6, 0.7, and 0.9 mm.

Figure 2(a) shows the *I-V* plot of a sample, which was measured with the voltage increasing from 0 V to a maximum value (V_max_) of 15 V with a scan rate of 0.1 V/s, and then the voltage decreased from V_max_ to 0 V at the same rate. In the voltage-up step (step 1), the current increases exponentially with the increasing of voltage, and then decreases to 0 A in the voltage-down step (step 2). However, current value in step 2 is higher than that in step 1, and a hysteretic loop is generated. These results indicate that the resistance of the sample varies with the history of the voltage loading, which is one of the main characteristics of a memristor and a memristive system. When the voltage scan rate is increased, the current and the loop area would decrease as shown in Figure 2(b), which is also a feature of memristive device.

Figure 2(c) shows the *I-V* loops obtained at different ambient temperatures. The resistance decreases with the increasing of the temperature. And a higher temperature causes a larger *I-V* loop area. Figure 2(d) shows the *I-V* characteristic of a series of samples with different thicknesses. The results indicate that, the current and *I-V* loop area increase with the decreasing of the thickness. 

**Figure 3:**
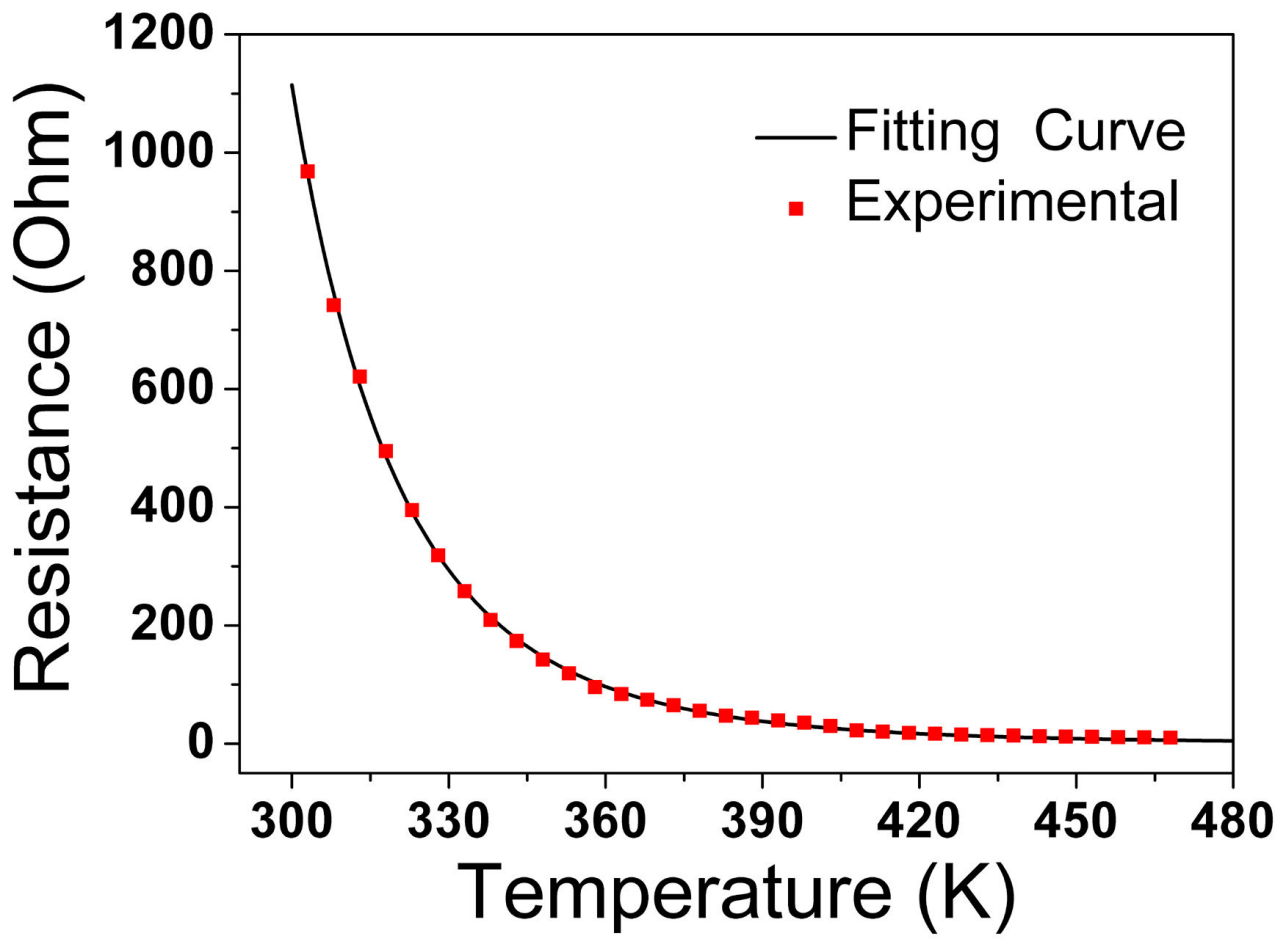
Plot of resistance as a function of temperature. The solid line is the fitting curve while the solid squares are experimental results.

*I-V* characteristics of the sample with Pt electrodes coated by vacuum sputter are found to be similar to those shown in Figure 2, indicating that the resistance switching behavior is not caused by the metal-NiO interface effect. In order to clarify the origin of the switching characteristics, the relationship between resistance and temperature was investigated and the results are shown in Figure 3. Resistance of the sample decreases with the increasing of the temperature leading to a NTC thermistor characteristic and resistance of an NTC thermistor can be characterized by [22] 

$$R_{T}=R_{0}\exp\left(B\left(\frac{1}{T}-\frac{1}{T_{0}}\right)\right)$$ (1)

where *T*is the temperature, *T*_0_is the initial temperature, *R*_*T*_and *R*_0_ are the resistances at the temperature of *T*and *T*_0_ respectively. *B*is a parameter which depends on material properties.

Eq. (1) was used to numerically simulate the measured results. The initial resistance and temperature were measured to be *R*_0_=1229 Ω and *T*_0_=298 K. The calculated resistance agrees well with the experimental result as shown in Figure 3 which indicates the NTC behaviors in NiO ceramics.

Heat generation and dissipation will occur when current travel through the sample, thus affects the temperature of the sample as shown in the following Eq. (2):

$$mC\frac{dT}{dt}=i^{2}R_{T}-h(T-T_{0})-\sigma_{b}(\varepsilon_{1}T^{4}-\varepsilon_{2}T_{0}^{4})$$ (2)

Where *m*is the weight of the sample, *C*is the heat capacity, *T*is the temperature, *t*is time, *i*is the current flowing through the sample, *R*_*T*_is the resistance at temperature of*T*, *h* is the convective heat transfer coefficient, and *T*_0_ is the initial temperature, *σ*_*b*_is Stefan-Boltzmann constant, *ε*_1_and *ε*_2_ are specific radiance of the sample and the surroundings, respectively. The first term in the right hand side of Eq. (2) describes the heat generation in the sample and the second and third terms in the right hand side of Eq. (2) give the heat dissipation in the sample, and the term in the left hand side describes the relationship between heat and the temperature variation. High voltage produces large current, and so heat generated will be more than that is dissipated. Thus heat accumulation will take place and this will lead to an increase in temperature. The reduction of the resistance of the sample occurs with the increasing in temperature. In the voltage-down step, the accumulated heat would dissipate, which leads to the temperature recovery, thus the resistance resets to its initial value, resulting in an *I-V* loop. Less heat is generated at high voltage scan rate, resulting in a smaller temperature rise and a smaller resistance change, and the area of the *I-V* hysteretic loop would reduce. 

A decrease in thickness accompanies weight reduction, leading to a larger temperature change when the accumulated heat is similar. Otherwise, the thinner samples exhibit lower resistance, resulting in an increase in current, hence, more heat will be generated than that in the thicker samples and the heat dissipated is nearly the same. In this case, the accumulated heat is more than that in the thicker samples and so enhanced temperature increase. Hence, the *I-V* loop area increases with the decreasing of the sample thickness. This phenomenon provides a proof that the memristive switching in this system is caused by bulk effect. 

**Figure 4:**
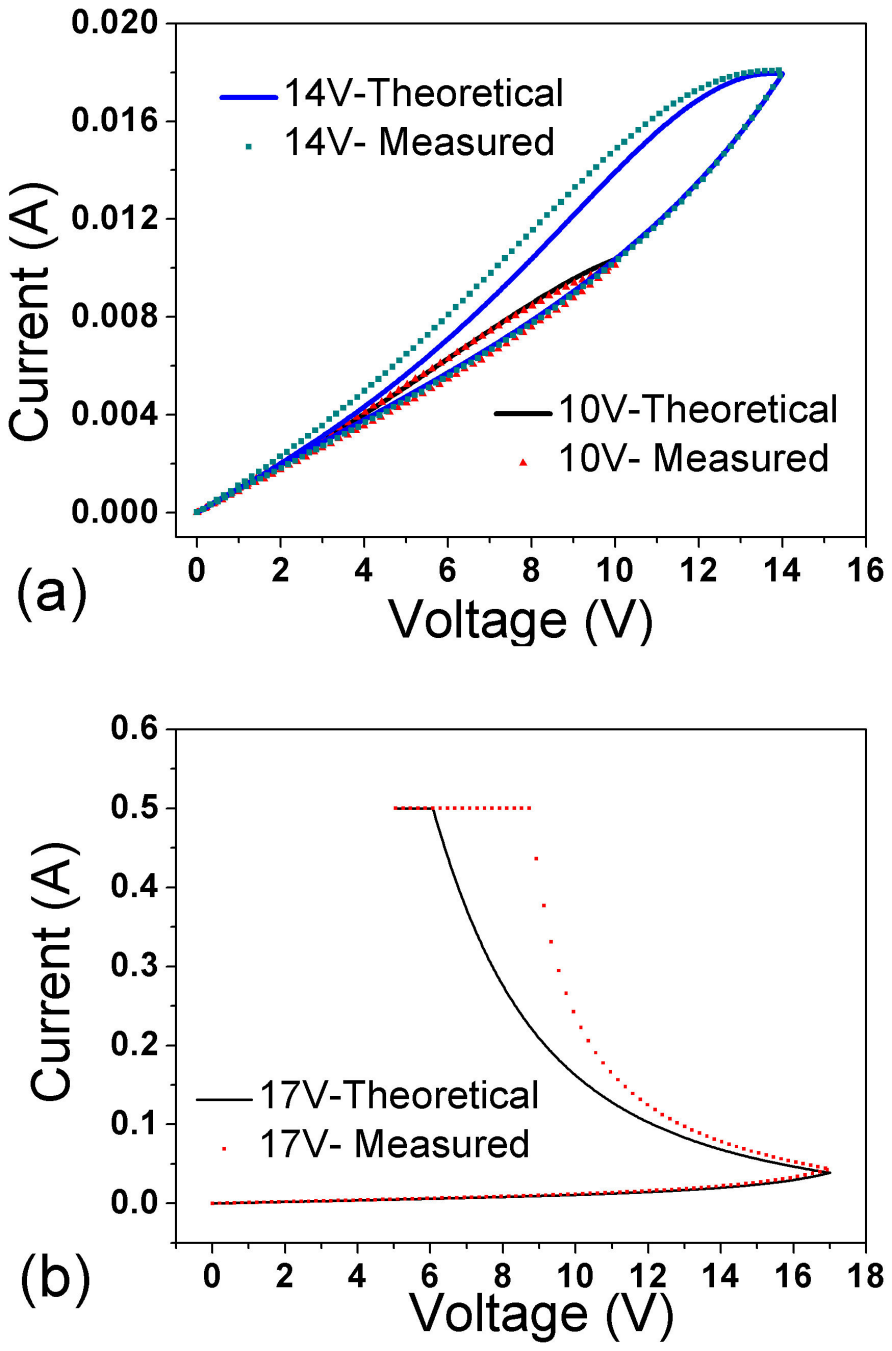
Theoretical curves (solide lines) and measured results (solide squares and solide triangles) of *I-V* characteristics at voltage scan rates of 1 V/s for: (a) V_max_=10 and 14 V, (b) V_max_=17 V.

As illustrated in Eq. (2), the heat generated is determined by the current through the sample. More heat is generated when the V_max_ gets higher, leading to the increasing of the temperature, thus the current and loop area will increase. At the very beginning of the voltage-down step, the current is still large enough, and the heat generated is more than that is dissipated, resulting in temperature increase and resistance reduction. This can be used to explain the fact that the current increase at the beginning of the voltage-down step before decreasing. This phenomenon is exaggerated when the V_max_ is increased to 17 V, during the voltage-down step, the current keeps rising untill reaching the instrument compliance limit as shown in Figure 4(b). The minor variation between the theoritical curve and measured results could be caused by the model simplification, where constant values are chosen for C and*δ*.

**Figure 5:**
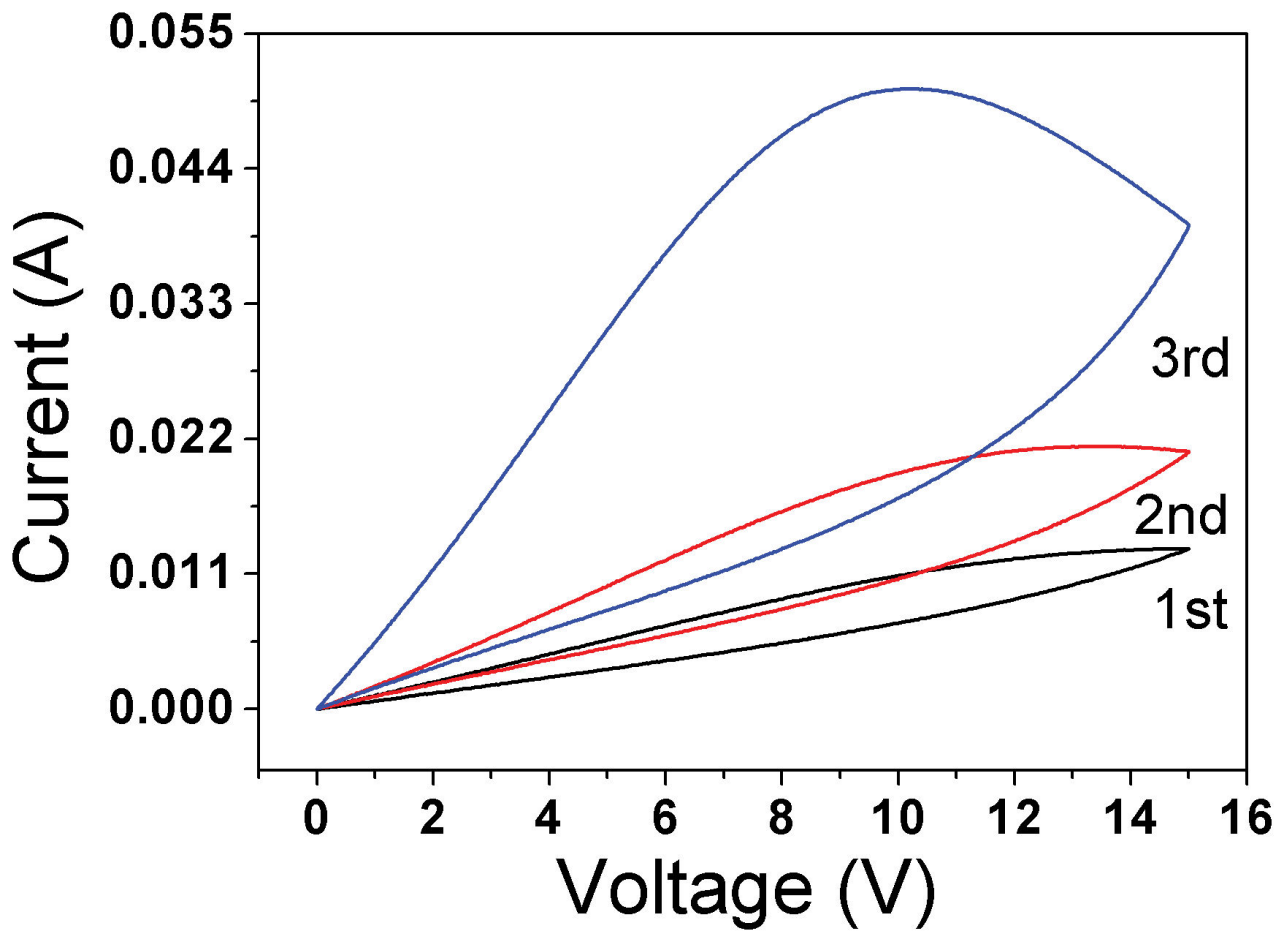
Three continuous cycles of *I-V* loops for sample encapsulated with expanded polyethylene with V_max_=15V at a voltage scan rate of 1 V/s.

For a better understanding of the mechanism, sample was encapsulated with expanded polyethylene to reduce the heat dissipation. As shown in Figure 5, current increases continuously in three voltage scan cycles with only a minor overlap. If the sample is left in the ambient condition for a long time, the *I-V* characteristics would go back to the initial state due to the heat dissipation and when the expanded polyethylene was changed to other materials with lower thermal conductivity or vacuum, an ideal memristive one-port is found and the resistance is a monotonically decreasing function of current. This makes it possible to control current-induced resistance change, which can be a potential solution to increase storage density. And, circuits with more functions can be built with fewer components. However, other NTC thermistor will exhibit similar behaviors in different voltage range.

## Conclusions

In summary, pinched hysteretic in *I-V* curves was observed in macroscopic bulk negative temperature coefficient nickel monoxide (NiO) ceramic material. The Ag/NiO/Ag cell exhibits a unipolar *I-V* loop that depends on the voltage scan rate, temperature, and size. A model depending on the negative temperature coefficient thermistor considering both the heat generation and dissipation was proposed to explain this phenomenon. This memristive mechanism would be significant in exploring memristive devices. 
